# Correlation analysis between renal anatomical factors and residual stones after an ultrasound-guided PCNL

**DOI:** 10.3389/fsurg.2023.1121424

**Published:** 2023-04-18

**Authors:** Shusheng Zhu, Yanpeng Fan, Xia Hu, Mingming Shao

**Affiliations:** ^1^ Department of Urology, Jining No.1 People’s Hospital, Jining, China; ^2^ Department of Urology, The First Hospital of Jilin University, Changchun, China; ^3^ Department of Geriatrics, Jining No.1 People’s Hospital, Jining, China

**Keywords:** percutaneous nephrolithotomy, renal anatomical, residual stones, SFR, ultrasound-guided

## Abstract

**Introduction:**

To predict the factors of residual stones after percutaneous nephrolithotomy (PCNL) by analyzing the characteristics of the renal anatomical structure in intravenous urography, so as to make a reasonable operation plan, reduce the risk of residual stones in PCNL, and improve the stone-free rate (SFR).

**Methods:**

A retrospective study was performed between January 2019 and September 2020 for patients treated with PCNL. According to the results of a kidney ureter bladder review after PCNL, 245 patients were divided into a residual stone group (71 patients, stone size >4 mm) and a stone-free group (174 patients, stone size ≤4 mm). An independent sample *t*-test was used to analyze the age, the length and width of channel calices, the angle between the channel calices and the involved calices, and the length and width of the involved calices. The gender, the channel types, the number of channels, the degree of hydronephrosis, and the number of involved calices were analyzed by using the chi-square test. A score of *p *< 0.05 was considered statistically significant. At the same time, logistic regression analysis was carried out to explore the independent influencing factors of the SFR after PCNL.

**Results:**

A total of 71 patients developed residual stones after surgery. The overall residual rate was 29.0%. The width of the channel calices (*p *= 0.003), the angle between the channel calices and the involved calices (*p *= 0.007), the width of the involved calices (*p *< 0.001), the channel types (*p *= 0.008), and the number of involved calices (*p *< 0.001) were all significantly correlated with residual stones after PCNL. Logistic regression analysis showed that the width of the channel calices (*p *= 0.003), the angle between the channel calices and the involved calices (*p *= 0.012), the width of the involved calices (*p *< 0.001), the channel types (*p *= 0.008), and the number of involved calyces (*p *< 0.001) were all independent influencing factors of the SFR after PCNL.

**Conclusion:**

A larger caliceal neck width and angle can reduce the risk of residual stones. The more calyces that are involved, the higher the risk of residual stones. There was no difference between F16 and F18, but F16 had a higher SFR than F24.

## Introduction

Percutaneous nephrolithotomy (PCNL) is considered the first choice of treatment for large (≥2 cm) and complex renal calculi (1, 2). However, it remains a challenge to predict whether PCNL can completely remove stones before surgery. At present, many scoring systems have been designed to predict the results of PCNL, such as Guy's scoring system (GSS) (3), the Clinical Research Office of the Endourological Society (CROES) nomogram (4), S.T.O.N.E. nephrolithometry (5), and Seoul National University Renal Stone Complexity (S-ReSC) score (6).

GSS classified the complexity of PCNL into four grades (I, II, III, and IV) according to the imaging characteristics of patients (3, 7). The S.T.O.N.E. score system was devised on the basis of five variables from non-contrast CT (NCCT), namely, stone size, tract length, degree of obstruction /hydronephrosis, number of involved calices, and stone density/Hounsfield units (5). The CROES nomogram was designed on the basis of an evaluation of six variables, namely, stone burden, stone location, initial treatment, staghorn stone, stone number, and annual operation volume. The scores of each variable could be added to obtain the total score and the corresponding stone clearance rate. The CROES nomogram could be divided into four grades (grade 1: 0–100, grade 2: 101–150, grade 3: 151–200, and grade 4: 201–350) (4). The calculation method of the S-ReSC score system depended only on the location of the stone, which turned out to be a simple process for the classification of the complexity of the disease. The researchers reasoned that the location of the stone was an important factor influencing surgery and therefore designed a 9-point system (low risk = 1–2 points, medium risk = 3–4 points, high risk = 5–9 points) (8).

However, the renal anatomy was not involved in the four scoring systems. In this study, we compared several renal anatomic factors associated with PCNL outcomes and studied the possible predictors of the stone-free rate (SFR) after PCNL.

## Clinical data and methods

**Figure 1:**
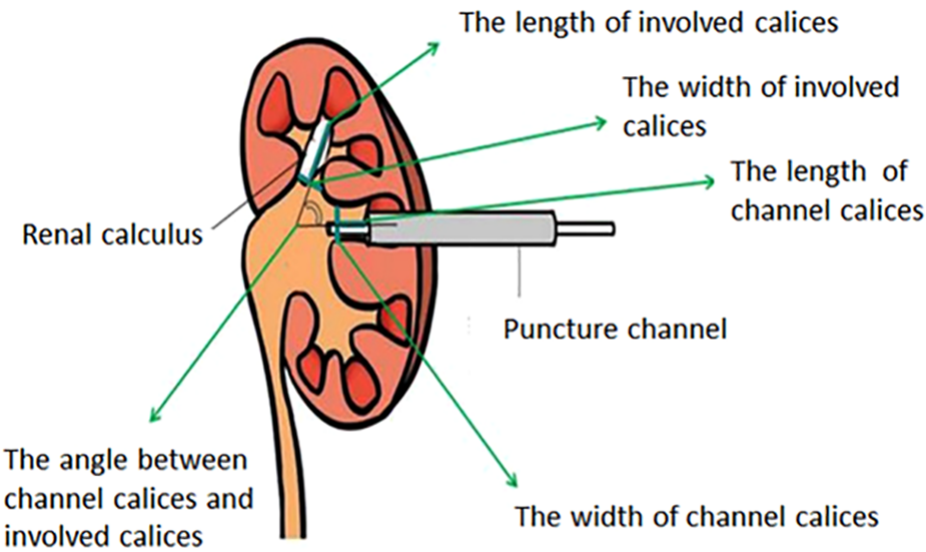
All anatomical data were measured on IVU.

A total of 245 renal calculi patients [152 male and 93 female, mean age: 52 years (range: 23–76)] were retrospectively reviewed from January 2019 to September 2020 in our hospital. Demographic, perioperative, and anatomical data for all patients were retrospectively collected, and the data included age, gender, the length and width of channel calices, the angle between channel calices and involved calices, the length and width of the involved calices, the channel types, the number of channels, the degree of hydronephrosis, and the number of involved calices (Table 1). All patients underwent NCCT and intravenous urography (IVU) before surgery, and a kidney ureter bladder (KUB) examination was performed within 1 week after operation. All anatomical data were measured on IVU (Figure 1). The residual stone group was defined in terms of residual fragments ≥4 mm on the KUB. If the stones contain multiple calices, the mean value of the relevant data should be taken, and in this study, patients with renal calculi with the same caliceal puncture were excluded. When analyzing the angle between the involved calyces and the channel calyces, and the length and width of the involved calyces, stratified analysis was used, and simple renal pelvis stones were excluded from the analysis. All surgeries were performed by the same surgeon.

**Table 1:** Clinical characteristics of study patients.

Variables	*n*/Total	Rate (%)
Gender		
Male	152/245	62.04
Female	93/245	37.96
Age	52.22 ± 11.58	–
Channel calices’ length (mm)	18.16 ± 7.05	–
Channel calices’ width (mm)	8.71 ± 3.28	–
Involved calices’ length (mm)	16.97 ± 6.54	–
Involved calices’ width (mm)	9.02 ± 3.21	–
Angle (°)	63.13 ± 47.56	–
The channel types		
F24	90/245	36.73
F18	58/245	23.67
F16	97/245	39.60
The number of channels		
1	240/245	97.96
2	3/245	1.22
3	2/245	0.82
The degree of hydronephrosis		
No/mild	215/245	87.76
Medium	19/245	7.76
Severe	11/245	4.48
The number of involved calices		
Pelvic stone	50/245	20.41
One calyx	90/245	36.73
Two calyces	64/245	26.12
≥Three calyces	41/245	16.74

## Ultrasound-guided PCNL procedure

**Figure 2:**
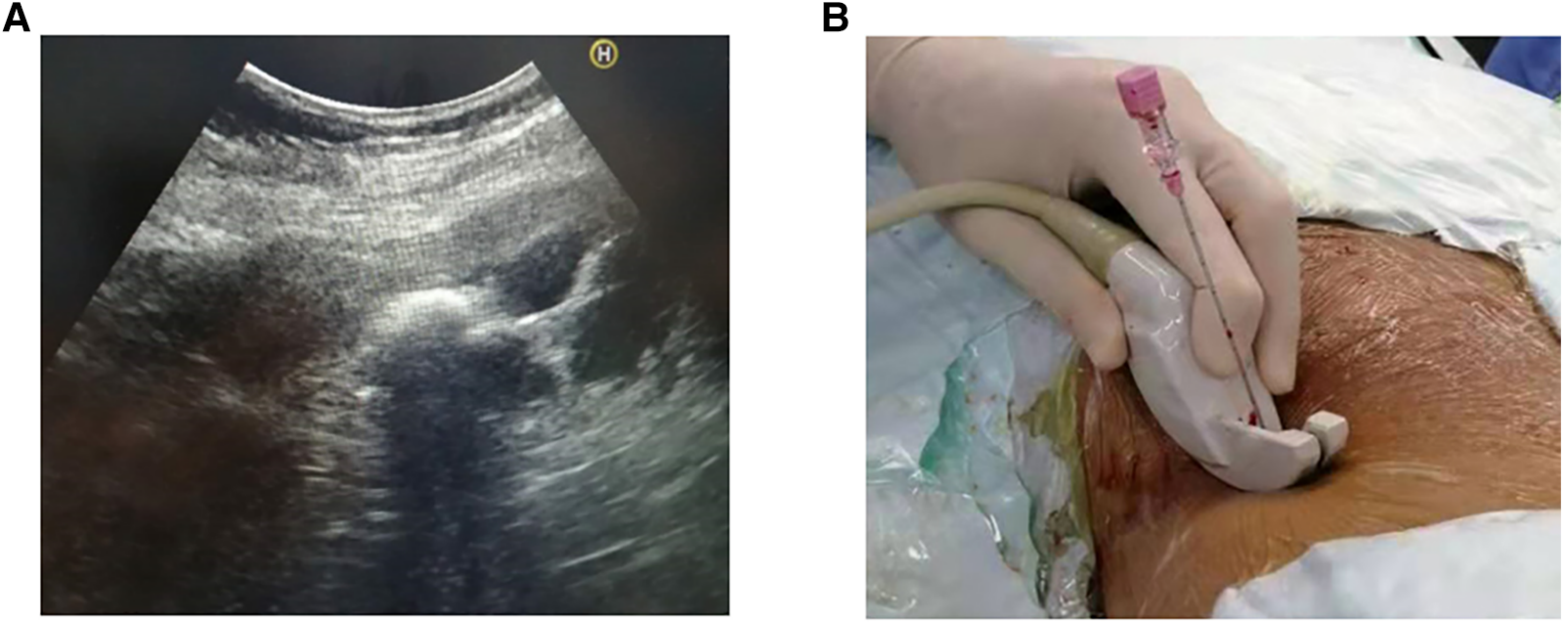
Ultrasonography guided localization and puncture.

**Figure 3:**
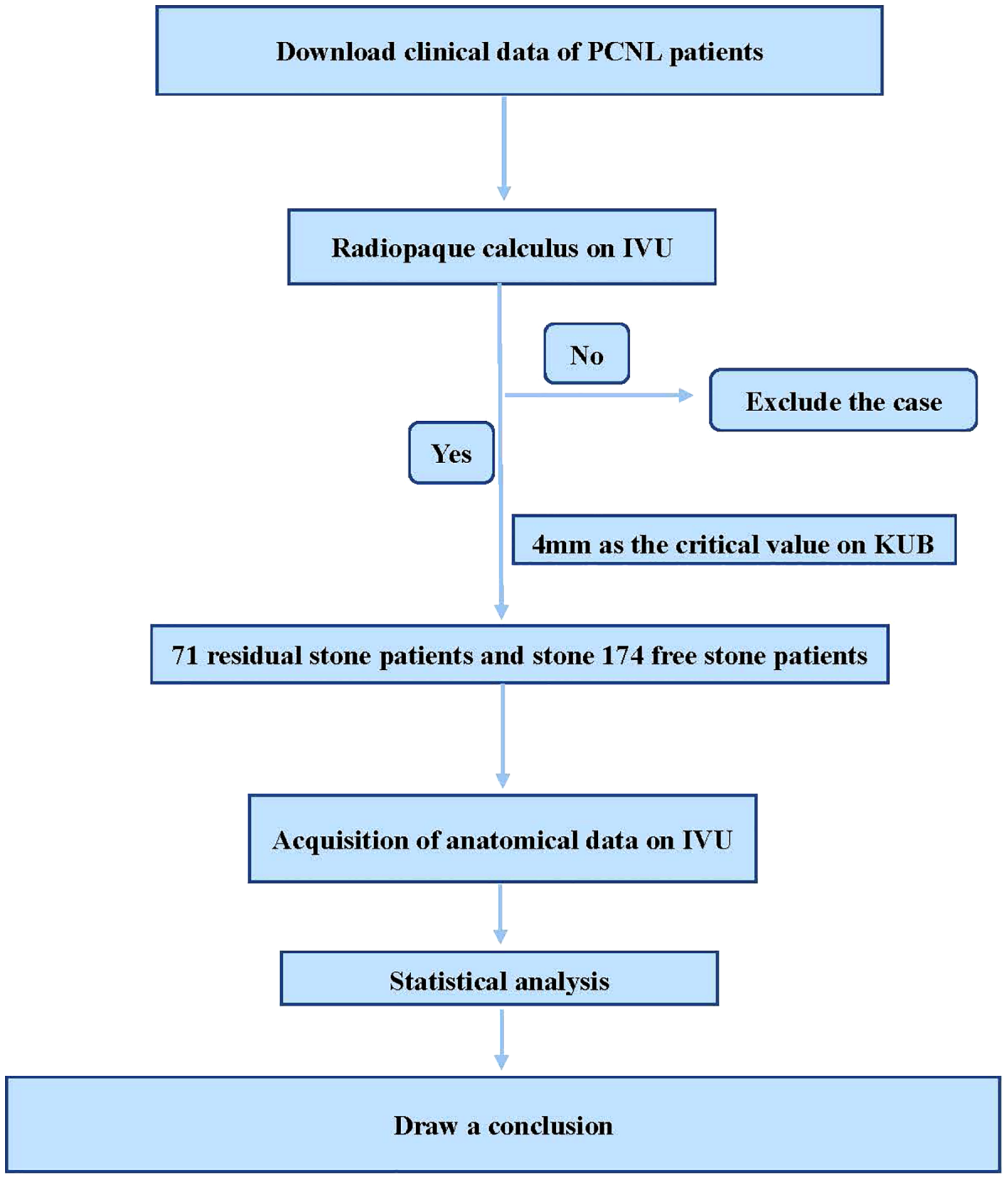
The flow chart of the study.

With the patient under general anesthesia, all operations were performed on the fluoroscopy table (Siemens, Berlin, Germany). The patient was initially placed in the lithotomy position, with a 5-Fr ureteral catheter retrograde attached to the renal pelvis under cystoscope. Then, the patient’s position was changed to prone. Under the guidance of ultrasound (Aloka 5, Tokyo, Japan, Figures 2A,B), the targeted calyx was selected according to the position of calculi and the degree of hydronephrosis. An 18-gauge coaxial puncture needle (COOK, Spencer, Ind., USA) was inserted into the predetermined calyx. Then, a 3.5-Fr floppy-tipped guide wire (Boston Scientific, Natick, MA, USA) was inserted into the renal pelvis using the X-Force Nephrostomy Balloon Dilation Catheter (Bard, Covington, GA, USA) to establish a working channel. Then, a 20-Fr nephroscope (Karl Storz, GmbH, Tuttlingen, Germany) was inserted into the renal pelvis through the working channel. The Cybersonics Double-Catheter System (Gyrus/ACMI, Southborough, MA, USA) was used for performing lithotripsy. Finally, a clamped 20-Fr modified Foley catheter was placed and was opened after 1 day. If no complications occur approximately 3 days after the operation, the catheter should be removed. Moreover, we regularly inserted the double J tube and removed it 1 month after the operation. The flow chart of the study is shown in Figure 3.

### Statistical analysis

The two groups were compared in terms of demographic, perioperative, and anatomical data using the Student’s *t*-test for obtaining continuous data and the chi-square test was used obtaining for categorical data. The significant factors in univariate analysis were analyzed by using logistic regression analysis to determine the independent influencing factors of the residual stones. The Statistical Product and Service Solutions for Windows (versions 22.0, SPSS, Inc., Chicago, IL, USA) was used for statistical analysis. A value of *p* < 0.05 was considered statistically significant.

## Results

We retrospectively compared 71 patients in the residual stone group vs. 174 patients in the stone-free group. There were no significant differences in age and gender between the two groups (Table 2). The length of the involved calyces was longer in the stone-free group (17.30 ± 5.68 vs. 16.39 ± 7.85 mm) than in the residual stone group, but it was not statistically significant (*p* = 0.356). The length of the channel calices was longer in the residual stone group (18.83 ± 7.76 vs. 17.89 ± 6.74 mm) than in the other group, but it was not statistically significant (*p* = 0.343). There was no significant difference in the number of tracts (*p* = 0.280) and the degree of hydronephrosis (*p* = 0.706) between the two groups (Table 2). The stone-free group had significantly wider channel calices (9.11 ± 3.41 vs. 7.74 ± 2.74 mm, *p* = 0.003), involved calices (8.84 ± 3.09 vs. 6.56 ± 2.89 mm, *p* < 0.001), and angle (69.99 ± 52.59 vs. 50.87 ± 33.94°, *p* = 0.007). There were significant differences in channel types (*p* = 0.008) and the number of involved calices (*p* < 0.001) between the two groups (Table 2).

**Table 2:** Statistical analysis of risk factors for residual stones after PCNL.

Variables	Residual stones	Stone free	*p*
Age	51.65 ± 12.11	52.46 ± 11.38	0.620^a^
Involved calices’ length	16.39 ± 7.85	17.30 ± 5.68	0.356^a^
Involved calices’ width	6.56 ± 2.89	8.84 ± 3.09	<0.001^a^
Channel calices’ length	18.83 ± 7.76	17.89 ± 6.74	0.343^a^
Channel calices’ width	7.74 ± 2.74	9.11 ± 3.41	0.003^a^
Angle	50.87 ± 33.94	69.99 ± 52.59	0.007^a^
Gender			0.783^b^
Male	45	107	
Female	26	67	
The channel types			0.008^b^
F24	36	54	
F18	10	48	
F16	25	72	
The number of channels			0.280^b^
1	68	172	
2	2	1	
3	1	1	
The degree of hydronephrosis			0.706^b^
No/mild	63	152	
Medium	6	13	
Severe	2	9	
The number of involved calices			<0.001^b^
Pelvic stone	1	49	
One calyx	9	81	
Two calyces	28	36	
≥Three calyces	33	8	

Data are shown as mean ± standard deviation.

^a^ Student's *t*-test.

^b^ Chi-square test.

Logistic regression analysis showed that the width of the channel calices (*p *= 0.003), the angle between the channel calices and the involved calices (*p* = 0.012), the width of the involved calices (*p *< 0.001), the channel types (*p* = 0.008), and the number of involved calyces (*p *< 0.001) were the independent influencing factors of the SFR after PCNL (Table 3).

**Table 3:** The results of logistic regression analysis.

Variables	*B*	SE	*p*	OR	95% CI	
					Upper	lower
Involved calices (≥3)						
Pelvic stone	−19.954	40,192	1.000	<0.001	–	–
One calyx	3.895	0.062	<0.001	46.862	13.913	157.836
Two calyces	1.758	0.570	0.002	5.798	1.896	17.732
Involved calices’ width	0.372	0.091	<0.001	1.451	1.213	1.736
Angle	0.013	0.005	0.010	1.013	1.003	1.023
Channel calices’ width	0.240	0.079	0.003	1.271	1.088	1.486
The channel types (F16)						
F24	−1.176	0.521	0.024	0.309	0.111	0.857
F18	0.399	0.611	0.513	1.491	0.451	4.934

## Discussion

PCNL was first introduced in 1976 (9). Nowadays, PCNL is the first-line treatment for large and complex renal stones (1, 10). However, some patients have residual stones, accounting for 24%–44% of postoperative patients (11). Because the residual stones may continue to grow, leading to infection and obstruction, it is of urgent importance for urologists to achieve an SFR. Meanwhile, there is a need for providing a model for preoperative patient counseling and standardized scoring system for the prediction of the SFR. At present, there are four scoring systems for predicting residual stones after PCNL, namely, S.T.O.N.E., GUY, CROES, and S-ReSC Scoring Systems (8). However, these scoring systems do not consider the influence of renal anatomical factors on the outcomes. To the best of our knowledge, the current study may be the first of its kind to study the influence of anatomical factors on residual stones after PCNL.

Historically, PCNL has been conducted under fluoroscopic guidance (12). However, the most significant disadvantage of fluoroscopy is the ensuing radiation exposure (13, 14). In previous studies, scholars have demonstrated the advantages of ultrasound-guided renal puncture and its superiority over fluoroscopy. It reduces both the cost and the radiation exposure of the patients (15, 16). Today, a combined approach of fluoroscopy and ultrasound is being increasingly used to overcome the challenges associated with renal punctures (17).

This study found that out of 245 patients with renal stones, 71 (29.0%) had residual stones after PCNL. The gender and age differences were small and were not predicted to affect the outcomes. The length of the channel calyces and involved calyces had no effect on the PCNL results. Because the length of the working channel in PCNL can reach up to 20 cm as long as the angle and calyceal neck width are appropriate, the operation channel can reach any position of the calyces. As the study was a retrospective one, the selection of the number of channels was influenced by the surgeon's preference. In our series, surgeons prefer single-channel lithotripsy, which, however, will give rise to the possibility of producing biased results. As the surgical assistant injected 0.9% of sodium chloride into the ureteral catheter during puncture positioning to create an artificial hydronephrosis, the question whether the patients had hydronephrosis during the course of their disease has little bearing on the results of PCNL. There were significant differences in the number of involved calices (*p* < 0.001) between the two groups. The number of calyces involved basically represents the complexity of the renal stone. The complexity of the stones increased with the number of calyces involved. Chen et al. reported that the residual rate of renal complex stones after PCNL is approximately 29.6% (18), which is consistent with our result. At the same time, the angle is a very important factor that must be factored-in during the operation, because a wrong angle will prove disastrous for the surgeon. If the calices have enough width, and the angle is greater, the stone removal rate will be higher. When there are parallel renal calyces, single-channel puncture is difficult to achieve in stone removal. A frantic effort to get the angle right during lithotripsy will only increase the complications associated with the procedure. In such situations, increasing the size of the puncture channel is the best option. However, this study showed that there was no correlation between the number of puncture channels and the SFR, which is an obvious discrepancy. This result may be related to the question of to what extent our center is technically equipped. Mini-PCNL (F16) had a significantly higher SFR than standard-PCNL (F24). However, there was no significant difference in the SFR between F16 and F18. According to a research conducted by ElSheemy et al. Mini-PCNL could reduce complications and hospital stay and most of the steps in this procedure could be performed in a tubeless way (19). In the anatomy of the kidney, it is extremely important to evaluate the width of the calyceal neck before PCNL. Only a wider renal calyce is suitable for puncture, which plays a highly important role in reducing postoperative complications. Using a larger nephroscope to puncture a narrower calyceal neck will only increase the risk of bleeding (20). In keeping with the above, we recommend the use of Mini-PCNL to puncture the wider calyces as the operation platform, which will greatly help in the prognosis of patients.

However, there are several limitations in this study that should be noted. First, we used the IVU method to study renal anatomical factors instead of the more accurate 3D-CT image reconstruction, which may impact the analysis of the measured data. Second, we used a retrospective research method rather than a prospective study, and therefore, a possible bias in the research results cannot be ruled out. Third, this finding may have some bias due to the fact that the F16/18 is mainly used for stones of smaller size. So, the chances of a higher SFR will be greater. With the same stone volume, the use of F24 may Úlso result in better clearance.

## Conclusion

A larger caliceal neck width and angle may reduce the risk of residual stones. The more the involved calyces, the higher the risk of residual stones. There was no difference between F16 and F18, but F16 had a higher SFR than F24.

## Data Availability

The original contributions presented in the study are included in the article/Supplementary Material; further inquiries can be directed to the corresponding author.
